# Wait-times benchmarks for risk-based prioritization in transcatheter aortic valve implantation: a simulation study

**DOI:** 10.1093/ehjqcco/qcae059

**Published:** 2024-07-19

**Authors:** Rafael N Miranda, Peter C Austin, Stephen E Fremes, Mamas A Mamas, Maneesh K Sud, David M J Naimark, Harindra C Wijeysundera

**Affiliations:** Institute of Health Policy, Management and Evaluation, University of Toronto, Toronto M5T 3M6, Canada; Institute of Health Policy, Management and Evaluation, University of Toronto, Toronto M5T 3M6, Canada; ICES, Toronto M4N 3M5, Canada; Institute of Health Policy, Management and Evaluation, University of Toronto, Toronto M5T 3M6, Canada; ICES, Toronto M4N 3M5, Canada; Temerty Faculty of Medicine, University of Toronto, Toronto M5S 1A8, Canada; Schulich Heart Program, Sunnybrook Health Sciences Centre, University of Toronto, Toronto M4N 3M5, Canada; Keele Cardiovascular Research Group, School of Medicine, Keele University, Stoke-on-Trent ST5 5BG, UK; ICES, Toronto M4N 3M5, Canada; Schulich Heart Program, Sunnybrook Health Sciences Centre, University of Toronto, Toronto M4N 3M5, Canada; Institute of Health Policy, Management and Evaluation, University of Toronto, Toronto M5T 3M6, Canada; Temerty Faculty of Medicine, University of Toronto, Toronto M5S 1A8, Canada; Institute of Health Policy, Management and Evaluation, University of Toronto, Toronto M5T 3M6, Canada; ICES, Toronto M4N 3M5, Canada; Temerty Faculty of Medicine, University of Toronto, Toronto M5S 1A8, Canada; Schulich Heart Program, Sunnybrook Health Sciences Centre, University of Toronto, Toronto M4N 3M5, Canada

**Keywords:** Transcatheter aortic valve implantation, TAVI, Health policy, Wait-times, Waitlist

## Abstract

**Background:**

Demand for transcatheter aortic valve implantation (TAVI) has increased in the last decade, resulting in prolonged wait-times and undesirable health outcomes in many health systems. Risk-based prioritization and wait-times benchmarks can improve equitable access to patients.

**Methods and results:**

We used simulation models to follow-up a synthetic population of 50 000 individuals from referral to completion of TAVI. Based on their risk of adverse events, patients could be classified as ‘low-’, ‘medium-’, and ‘high-risk’, and shorter wait-times were assigned for the higher risk groups. We assessed the impacts of the size and wait-times for each risk group on waitlist mortality, hospitalization, and urgent TAVIs. All scenarios had the same resource constraints, allowing us to explore the trade-offs between faster access for prioritized patients and deferred access for non-prioritized groups. Increasing the proportion of patients categorized as high-risk, and providing more rapid access to the higher-risk groups achieved the greatest reductions in mortality, hospitalizations and urgent TAVIs (relative reductions of up to 29%, 23%, and 38%, respectively). However, this occurs at the expense of excessive wait-times in the non-prioritized low-risk group (up to 25 weeks). We propose wait-times of up to 3 weeks for high-risk patients and 7 weeks for medium-risk patients.

**Conclusion:**

Prioritizing higher-risk patients with faster access leads to better health outcomes, however this also results in unacceptably long wait-times for the non-prioritized groups in settings with limited capacity. Decision-makers must be aware of these implications when developing and implementing waitlist prioritization strategies.

## Graphical Abstract

**Graphical Abstract:**
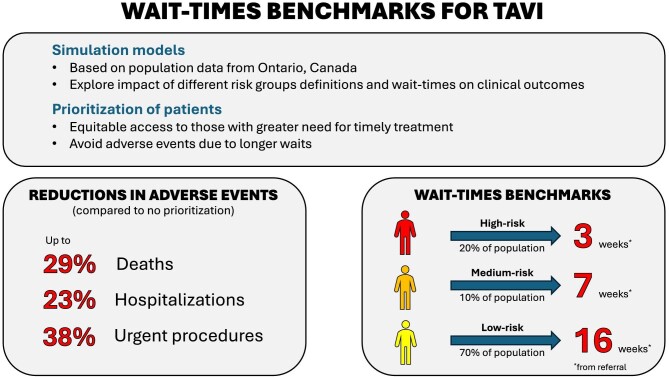

Key Learning Points What is already known:

- Indications for transcatheter aortic valve implantation (TAVI) have been expanded in the last decade, increasing the demand for the treatment. Proper waitlist management becomes paramount, specially in health systems with limited capacity.

What this study adds:

- We used decision-analytic models and a prediction tool to define risk group classifications and wait-time benchmarks that can improve health outcomes whilst keeping reasonable wait-times for all patients.
- Our wait-times and group definitions can be used by other jurisdictions as part of their waitlist management strategies for patients referred for TAVI, providing equitable and timely access to treatment.

## Introduction

Transcatheter aortic valve implantation (TAVI) for the treatment of severe aortic stenosis (AS) has emerged as the standard of care for inoperable and high-surgical risk patients, and as an alternative for medium- and low-surgical risk patients; this has translated to an exponential growth in demand.^1^ In many health systems, the expansion in the capacity to perform TAVIs has been insufficient to match the expansion in referrals for the procedure, leading to increasing wait-times.^2,3^

With such pressures on capacity resulting in restricted access to treatment—in particular for a condition such as AS which has a very poor prognosis when untreated^4^—the issue of adverse events while waiting must be balanced against equitable access. Often, these two factors are in tension. A possible solution to address wait-time morbidity and mortality is prioritizing patients who are at higher risk of adverse events, which can be identified based on clinical and sociodemographic factors, disease severity and comorbidities.^5,6^ Risk-based prioritization may improve the efficiency of the system by reducing the occurrence of avoidable hospitalizations or the need of urgent procedures.^5^ In fact, prioritization in healthcare is ubiquitous and its use as a waitlist management strategy has been well established across different areas, from public health interventions to critical procedures such as organ transplants.^7,8^

Although the rationale for the development of a prioritization strategy is often clear, less evident is how to consider and incorporate in the strategy factors that can impact the effectiveness of the strategy in minimizing adverse events in the population of interest. As certain groups are given faster access to treatment, others will have to wait longer, which increase the time they remain at risk of complications of disease or deterioration in health; at some point the benefits achieved in the prioritized group can be outweighed by increased complications in the non-prioritized groups. Some of the factors that can impact health outcomes and wait-times in the non-prioritized groups include the definition of the criteria used for classification, the number of categories that patients can be classified into, the progression of the disease and characteristics of the population. Also important during the development of a prioritization strategy is the consideration of the system capacity to offer treatment, as it may limit the extent to which prioritization can actually be possible without leading to extremely long wait-times in the non-prioritized groups.

In this study, we explored how these factors and the resource constraints impact the outcomes of a prioritization strategy for patients with severe AS referred to TAVI, using a validated tool that predicts patients’ risks of adverse events while in the waitlist, the Canadian TAVI Triage Tool (CAN3T).^9^ Our objective was to define risk groups size and wait-time benchmarks that reduce the number of adverse events in the population while ensuring reasonable wait-times for all patients, using the example of Ontario, Canada.

## Methods

### Study design and model description

This study used an individual-level (microsimulation) model, which simulated a waitlist of 50 000 individuals referred for TAVI in weekly cycles. The model simulated a closed cohort, i.e. there were no pre-existing patients on the waitlist nor were they added later. A schematic of the model can be found in Supplementary material online, *Figure S1*. While waiting for TAVI, patients could be hospitalized or die. Urgent (non-elective) TAVIs were incorporated in the model as a proportion of the individuals who were hospitalized in a cycle and required an unscheduled procedure. A distribution of predicted risk for adverse events based on the Ontario population was used to sample, for each simulated individual, a predicted risk of mortality, and hospitalization. This risk was then used to classify the individual as high, medium or low risk, based on thresholds that varied depending on the size of the risk group (e.g. when the size of the high-risk group was 10% of the population, the 90th percentile of the distribution of predicted risks was used as the threshold for the classification). A wait-time for TAVI was assigned to the individual based on the risk classification, with shorter wait-times for the higher-risk groups, simulating their prioritization for the procedure. The assigned wait-times and the size of each risk group varied by scenario analysed. To incorporate the deterioration in health that occurs with longer wait-times, the hazards of death and hospitalization increased every cycle, and were modelled using Weibull distributions. The simulated patients remained in the model until they died or underwent TAVI.

An important element of the model is resource constraints, as the number of procedures performed is limited and providing faster access to a group of patients must result in longer wait-times for the non-prioritized group. To assess the effects of prioritization, isolated from changes in wait-times that can occur over time due to variations in referrals or procedures performed, we assumed a constant demand and capacity for TAVI. The constant capacity/resource constraint was modelled by keeping a constant average wait-time of 12.5 weeks (based on data from Ontario) for the procedure in all scenarios analysed. Therefore, the wait-times in the non-prioritized group were always calculated so as to satisfy the condition that the overall wait-time for the entire cohort, calculated as the average of the group-specific wait-times weighted by their sizes, was equal to 12.5 weeks.

### Data sources, input parameters, and calibration

Model input parameters and calibration targets can be found in Supplementary material online, *Table S1*. The main data source for this study was the CorHealth Ontario Clinical Registry, which has demographic, comorbidity, and procedural data for all centres in Ontario that perform TAVI. The registry was linked to other databases at ICES, allowing us to capture supplemental information on the population and events during the study period. The use of anonymized administrative data held at ICES without patient consent is permitted in Ontario based on section 45 of Ontario's Personal Health Information Protection Act, which does not require review by a Research Ethics Board. A cohort of patients referred for TAVI in ON, between April 2012 and March 2020 was used to derive the input parameters of our model, including the risk of adverse events and the average wait-time for TAVI.

Another data source for this study was the cohort from which the CAN3T was derived.^9^ Briefly, the CAN3T models predict the risk of adverse events in patients referred for TAVI, including all-cause mortality, all-cause hospitalization, and risk of an urgent procedure. The CAN3T models were applied to our study cohort, generating a distribution of predicted risks for each outcome of interest. These distributions were incorporated in our simulation models to vary the size of each risk group. For example, when the scenario had the high-risk group equal to 5% of the cohort, we used the 95th percentile of the distribution of the predicted risk at 12 weeks among the CAN3T cohort as the threshold to classify the simulated patients as high-risk. It follows that using a lower threshold (i.e. the 70th percentile of the distribution of predicted risk at 12 weeks), would result in a larger high-risk group (i.e. 30% of the cohort).

Calibration was performed for a modelled reference scenario which simulated the *status quo* in Ontario: no risk-based classification nor wait-time benchmarks were employed and simulated patients received elective TAVIs on a first-come, first served basis. Model input parameters that affected the probability of death, hospitalization, and urgent TAVI were adjusted in order that modelled outcomes were similar to actual values observed in the CorHealth registry during the 2019 fiscal year (Supplementary material online, *Table S1*). The calibration targets were defined based on the 3030 referrals and 1330 TAVIs that were recorded in Ontario for the 2019 fiscal year. Over this period, after the exclusion of 803 patients who were removed from the waitlist, 816 patients (36.6%) remained on the waitlist at the end of 2019, 81 patients (3.6%) died on the waitlist, 756 (33.9%) had at least one unplanned hospitalization, and 202 of the TAVIs performed (15.2%) were unscheduled procedures.

### Analysis

To assess the impact of a risk-based classification system and use of wait-time benchmarks, we simulated three scenarios. First, we modelled three risk groups with thresholds of the predicted risk distribution selected such that the proportion of patients classified as high- and medium-risk varied from 5% to 30% of the cohort (each in increments of 5%), and with the low-risk group consisting of the remainder of the cohort. Second, we chose thresholds such that the three groups each constituted 1/3 of the cohort. Third, we analysed a scenario with only two-risk groups. Risk thresholds were chosen such that the size of the high-risk group varied between 5 and 30% and the low-risk group was the remainder of the cohort. The three scenarios were each compared to the reference scenario used for the calibration.

Across all scenarios, we set the following possible wait-times: 3, 4, or 5 weeks for high-risk patients, and for each high-risk wait-time, an incremental 2, 4, or 6 weeks for medium-risk patients. The wait-time for the low-risk group was selected such that the weighted average wait-time across groups was equal to 12.5 weeks. All 372 combinations of risk-group sizes and wait-times are summarized in *Table 1*. The results presented are averages across 100 simulation replicates for each combination with 50 000 patients per simulation replicate.

**Table 1:** Combinations of size and wait-times for each risk group

High-risk	Medium-risk	Low-risk
A. Proportion of cohort		
5–30%^a^	5–30%^a^	Remaining
33.33%	33.33%	33.33%
**High-risk**	**Medium-risk**	**Low-risk**
B. Wait-times (weeks)		
3–5	+2, +4, +6^b^	Calculated
	**High-risk**	**Low-risk**
C. Two risk groups		
Proportion	5–30%^a^	Remaining
Wait-time	3–5	Calculated

^a^In increments of 5%.

^b^In addition to the wait-time for the high-risk group.

Results for all the analyses are presented as heatmaps, which for each outcome, were coloured in the same scheme to facilitate comparisons among the three described above scenarios compared with the reference scenario. Analyses were conducted in TreeAge Pro (v. 2023 R2.0) and the R statistical environment (v. 4.3.2).

### Wait-times benchmarks

To derive wait-times benchmarks, we chose the combination of risk-groups sizes and wait-times that resulted in the best outcomes, while keeping a maximum wait-time of 16 weeks from referral for the non-prioritized group, which is consistent with the current Canadian Cardiovascular Society guidelines of 12 weeks from assessment for TAVI for stable patients.^10^

## Results

In the reference scenario, with no classification nor prioritization of patients for TAVI, our models predicted an all-cause waitlist mortality of 3.6%, all-cause waitlist hospitalization of 33.4%, and a proportion of patients undergoing urgent TAVIs of 15.2%.

### Impact of size and wait-times of higher risk groups on health outcomes

**Figure 1:**
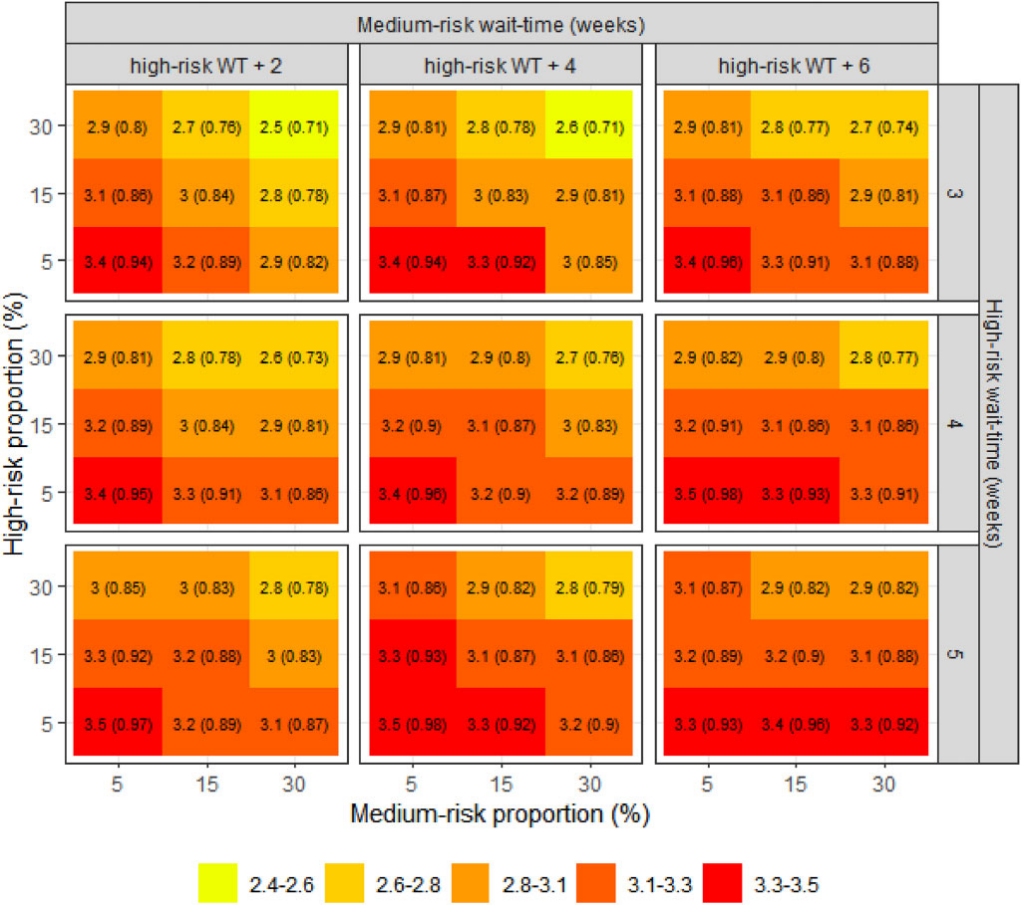
Impact of size and wait-times of the high- and medium-risk groups on mortality [% (RR)]. RR: relative risk compared to base-case scenario. WT, wait-time.

**Figure 2:**
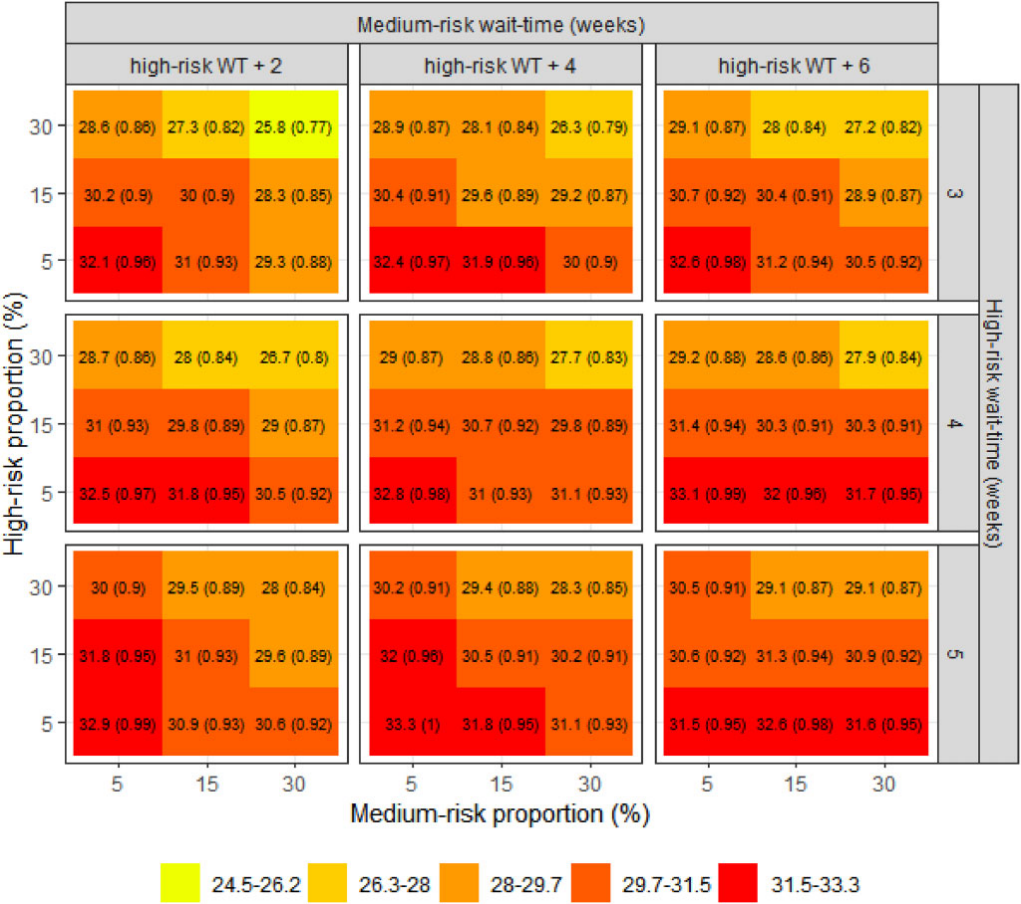
Impact of size and wait-times of the high- and medium-risk groups on hospitalizations [% (RR)]. RR: relative risk compared to base-case scenario. WT, wait-time.

**Figure 3:**
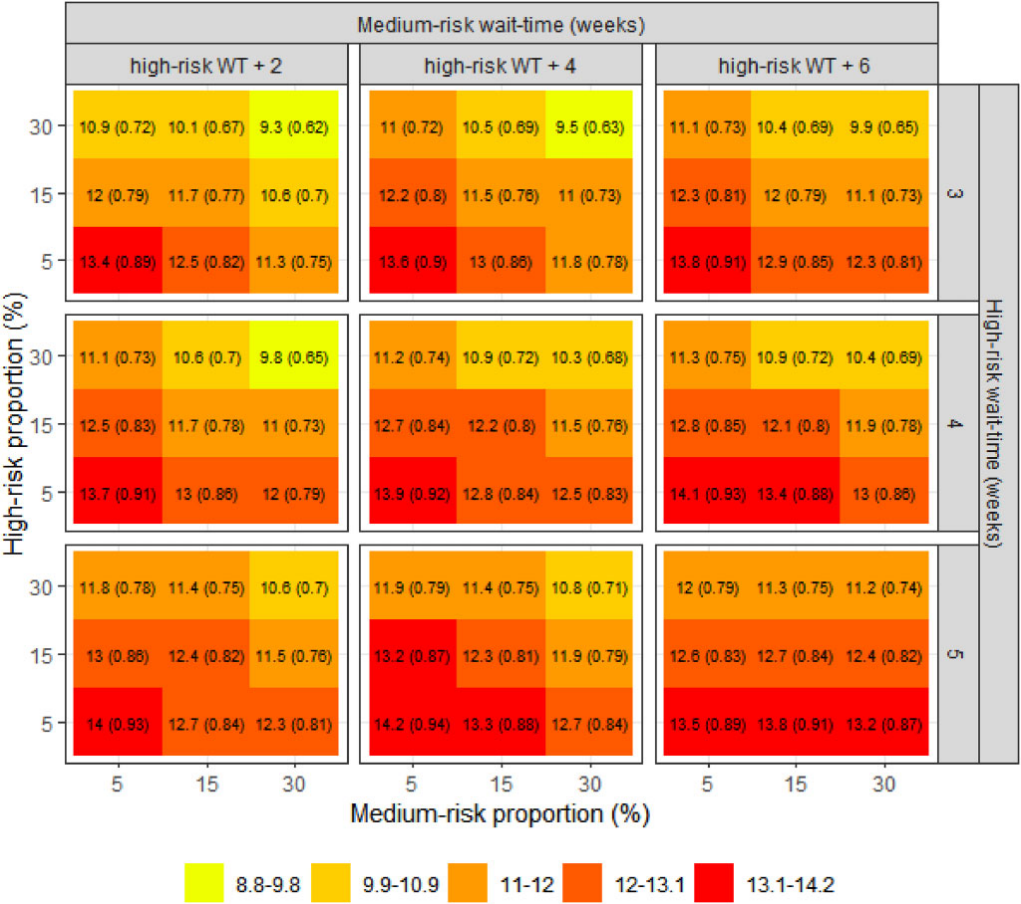
Impact of size and wait-times of the high- and medium-risk groups on proportion of urgent TAVIs [% (RR)]. RR: relative risk compared to base-case scenario. WT, wait-time.

*Figures 1*–*3* summarize the impacts of the group sizes and wait-times on mortality, hospitalization, and urgent TAVI, respectively. The isolated impact of the wait-times can be seen comparing the same quadrant between panels in the same row or column of the figures. The impact of modifying the wait-times for the high-risk group alone was greater than modifying the wait-time of the medium-risk group. For example, when high- and medium-risk groups were 30% of the cohort each (top-right quadrants of each panel), increasing the wait-times in the high-risk group increased the proportion of patients who underwent an urgent TAVI from 9.3 to 10.6% whereas increasing the medium-risk wait-time increased this proportion from 9.3 to 9.9%.

The isolated impact of the groups’ sizes can be seen comparing the quadrants within each panel of the figures. The impact of modifying the size of the high-risk group alone was greater than the impact of the modifying the size of the medium-risk group. This is particularly evident when wait-times for the prioritized groups were higher (bottom-right panel of the figures); e.g. increasing the size of the high-risk group decreased the proportion of patients who underwent urgent TAVIs from 13.5 to 12%, whereas increasing the size of the medium-risk group decreased this proportion from 12 to 11.2%.

**Figure 4:**
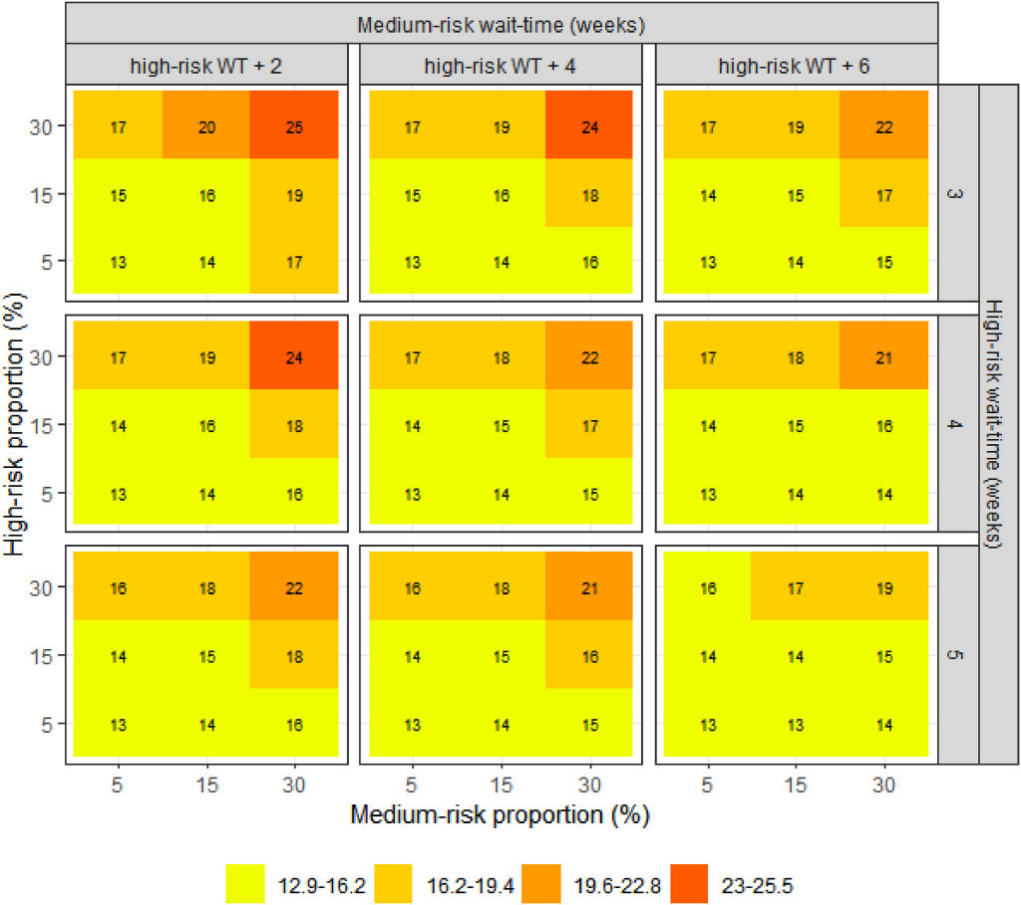
Impact of size and wait-times of the high- and medium-risk groups on wait-times in the low-risk group (weeks). WT, wait-time.

With a larger proportion of the cohort defined as high-risk and greater prioritization of this high-risk group with shorter wait-times, there were improved wait-time outcomes. The same trend was observed when we increased the size and shortened the wait-times for the medium-risk group. The lowest waitlist mortality was 2.5% (upper left pane of figures), corresponding to a relative risk (RR) of 0.71 compared with the reference scenario; the lowest hospitalization rate was 25.8% (RR = 0.77); and the lowest proportion of patients undergoing urgent TAVIs was 9.3% (RR = 0.62). These modelled optimal outcomes were observed when the high-risk group comprised 30% of the cohort, with a benchmark wait-time threshold of 3 weeks from referral; the medium risk group was 30% of the cohort with a wait-time benchmark of 5 weeks; and with the remaining 40% being classified as low-risk. The modelled risk proportions and wait-time benchmarks that produced optimal outcomes overall resulted in longer wait-times for the low-risk group at 25 weeks (*Figure 4*).

On the other hand, health outcomes had minimal improvement compared to status quo when the sizes of the high- and medium-risk groups were smaller (bottom panes of figures). The highest mortality was 3.5% (RR = 0.98), the highest hospitalization rate was 33.3% (RR = 1), and the highest proportion of patients undergoing urgent TAVIs was 14.2% (RR = 0.94). In this scenario, the high- and medium-risk groups consisted of 5% of the cohort, with the remaining 90% being low-risk; wait-times were 5 weeks for high-risk, 9 weeks medium-risk, and 14 weeks for the low-risk group.

### Sensitivity analysis

Results from the sensitivity analysis are provided in the supplement. Overall, compared to the main analysis, when the three risks groups had the same size, the improvement in health outcomes was greater, at the expense of a longer wait-time for the low-risk group. For example, in the combination with best outcomes mortality was 2.4% (RR 0.67, Supplementary material online, *Figure S2A*) but the wait-time for low-risk patients was 29.5 weeks (Supplementary material online, *Figure S2D*). The opposite was seen in the analysis with two risk-groups, with minor improvements in health outcomes and shorter wait-times in the low-risk group. In the combination with best outcomes, mortality was 2.9% (RR 0.8, Supplementary material online, *Figure S3A*) and wait-times in the low-risk group were 16.6 weeks (Supplementary material online, *Figure S3D*).

## Discussion

This study assessed how definitions of risk-groups in risk-based prioritization for waitlist management have important implications on the potential improvements on health outcomes and on the access of non-prioritized groups to an intervention. Using the example of Ontario, Canada, we found that the best outcomes were achieved when we increased the number of risk groups, used a low threshold to define the higher risk groups, and then provided faster access to these prioritized groups. However, based on current capacity, these improvements will result in unreasonable wait-times for the non-prioritized population. In settings or circumstances where the average wait-times are already long, overall system capacity must be increased so that improvements in access to prioritized groups do not occur at the expense of extremely delayed access for non-prioritized patients.

### Wait-times benchmarks

Based on the results from our study, we propose the following definitions of risk groups and wait-time benchmarks:

- a high-risk group consisting of the top 20% of population and a wait-time of 3 weeks from referral,
- a medium-risk group consisting of 10% of the population and wait-time of 7 weeks,
- the remaining population with a wait-time of 16 weeks from referral.

### Sensitivity analysis

The sensitivity analyses allowed us to assess the impact of the number of risk groups, and the impact of dividing a cohort into three groups of equal sizes. Compared to the results of our main analysis, when we divided the cohort into three groups with equal sizes we observed greater improvements on health outcomes, but this came at a higher cost for the non-prioritized patients as in none of the combinations the wait-times of the low-risk group were shorter than 16 weeks. Conversely, when we divided our cohort in two risk groups we found shorter wait-times in the low-risk group but also lower improvement in health outcomes. These results demonstrate that, specially when resources are limited, the proportion of the cohort being prioritized can result in extremely delayed access to the non-prioritized groups and therefore decision makers should be aware of the current context and system capacity during the development of a prioritization strategy. Furthermore, the stratification of patients into multiple risk categories can be a viable alternative to improve access to those at greater need while keeping reasonable wait-times for all patients, particularly when resources are scarce.

### Prioritization strategies and health equity

Prioritization strategies are designed to provide fair access to care,^11^ but fairness is a multi-dimensional concept that can be understood not only as faster access to those at higher need, but also as equitable opportunities to treatment. As seen in our study, these objectives may conflict with one another^12^ and the current supply and demand for a procedure will have a direct impact on the extent of improvement in health outcomes that can be actually achieved without causing unreasonable inequities in opportunities to care. Therefore, it is critical that not only these different principles and objectives are considered and balanced against each other, but also that decision-makers weigh them understanding the local context and the system resource constraints. These considerations are particularly important in contexts where there is limited opportunity for an expansion of services or a reduction in demand for a procedure.

An important aspect of prioritization frameworks is that there is no consensus on the inclusion or the weight of each criterion used for prioritization. Indeed, different criteria will reflect different values, needs and preferences of a particular community and setting.^13^ However, paramount to the prioritization system is the concept of equity—whether it is defined by clinical need, social justice or equality of opportunity. Some prioritization frameworks expand on the concept of equity beyond the clinical need of the patient, including non-clinical criteria such as impact on daily activities, ability to care for dependants and even historical and social factors.^11,14,15^ The time patients remain on the waitlist can also be included as a criterion to avoid excessively long wait-times for those in non-prioritized groups.^16^ Besides the goal of promoting equitable and fair access to care, prioritization systems should also be transparent, with clear and consistent criteria and definitions, and evidence-based, using scientific valid tools.^11,13^ Although there is limited evidence on the impact of prioritization strategies on health outcomes,^13^ there are examples showing that the benefits of prioritization as a waitlist management strategy go beyond the reduction in adverse events^17^ and include improvements to data quality and system efficiency.^11,13,18^

It is also noteworthy that prioritization strategies should be developed and implemented consistently and transparently in order to effectively reduce inequities in access to care. Although the implementation of the prioritization strategy ultimately occurs at the institutional level, its development should occur in the health system level and should include all stakeholders involved in the patient journey. If different prioritization schemes or criteria for prioritization are used by centres within the same health system, an additional source of inequitable access to treatment could arise based on the treatment centre.

### Impacts on clinical outcomes

Compared to the base-case scenario (i.e. the status quo), the outcome mostly impacted by prioritization was the proportion of patients undergoing urgent TAVIs (minimum RR of 0.62 vs. 0.71 for mortality and 0.77 for hospitalization). This occurred because the baseline distribution of the baseline risk of an urgent TAVI in our cohort was the most skewed among the outcomes. Therefore, even when the high-risk group had a small size (5%) it was able to capture a sample of the population with a baseline risk that is much greater than the overall risk of the population. This demonstrates that the prioritization of even a small group can result in more adverse events prevented, and decision-makers should be aware of when defining the outcomes for the prioritization strategy. Another criterion that can be used for prioritization is the severity of the outcome to be prevented, and in this case one could argue that mortality should whenever possible be the outcome of choice.

### Implementation and future directions

An important question that arises when using risk-based prioritization for waitlist management is how to define the risk groups, as these definitions will determine what proportion of the population will be considered low-, medium-, or high-risk. Decision-makers can define a set of criteria or use risk-prediction tools and calculators to classify patients into risk groups during medical assessment. One such tool, developed by our group specifically for patients referred for TAVI is the CAN3T calculator, which predicts the risk of adverse events at specific points in time after referral. In order to classify patients into risk groups with the CAN3T, it will be necessary to use numerical thresholds. In Supplementary material online, *Table S2* we provide the distribution of predicted risks in the population on which the CAN3T was derived, and in *Table 2*, we use the definitions of risk groups from this study to provide thresholds for the classification of patients using the CAN3T. For example, if the outcome used for classification is all-cause mortality, a patient should be considered high-risk if the predicted risk at 12 weeks is >3.7% (the 80th percentile of the distribution in our cohort if the high-risk group has a size of 20% of the population), and medium-risk if the predicted risk is between 3.7 and 3.3% (70th and 80th percentiles if the medium-risk group has a size of 10%).

**Table 2:** Thresholds for the definition of a high-risk group with 20% of the population and medium-risk group with 10% of the population using the CAN3T calculator

Outcome	Medium-risk threshold (%)^a^	High-risk threshold (%)^b^
All-cause mortality	3.3	3.7
All-cause hospitalization	35.5	38.4
Urgent TAVI	11.4	13.0

^a^70th percentile of the predicted risk distribution at 12 weeks.

^b^80th percentiles of the predicted risk distribution at 12 weeks.

Future research could benefit from our study and expand our understanding of critical parameters that should be considered when developing prioritization strategies. For example, including changes in demand for treatment was outside the scope of our study, but we understand that the TAVI landscape is dynamic and subject to increasing demand, whether by expansion of its indication or by an ageing population. This increasing demand for TAVI requires capacity for the procedure should also increase. Simulation models could be developed to incorporate temporal trends and changes in the clinical profile of patients with SAS and assist the proper planning of expansion of TAVI by predicting what be the necessary system capacity to ensure that every patient receives treatment in reasonable wait-times.

### Limitations

Our study must be interpreted in the context of its limitations. As a model-based study, its findings cannot be used as evidence of the effectiveness of prioritization in the reduction of adverse events in patients waiting for TAVI. Our models could not simulate some possible events that may occur during the patient journey from referral to procedure, such as reassessment of the patient and reclassification to a higher risk category, nor changes in demand or capacity for TAVI. Furthermore, we did not model the post-procedural period so it was not possible to assess any possible impacts on post-procedural outcomes. An important assumption used in our models was that the increase in the hazards of adverse events with longer wait-times follow a Weibull function, which may not hold in a real-life setting. If, e.g. deterioration in health is even more accelerated after long wait-times, non-prioritized groups can experience increased adverse events. Thus, we recommend that in settings where the implementation of a prioritization strategy would result in long wait-times for non-prioritized groups, these patients should be closely monitored for any changes in their functional status or disease severity.

Another limitation is that the performance of a prioritization strategy also depends on the performance of the tool used for prioritization. In our study, we used the CAN3T tool which has only achieved moderate performance in predicting adverse events and was validated on a sample from Ontario, Canada. Therefore, in a real-life setting and other jurisdictions, we would not expect improvements in health outcomes to the same extent as observed in our simulations. However, given that in all scenarios the prioritization achieved better outcomes compared to no prioritization, that there is an ethical rationale for the use of a prioritization system in waitlists, and that the implementation of a prioritization strategy can bring improvements beyond clinical outcomes, we believe our findings can be an important source of information for decision-makers and our recommendations can improve TAVI waitlist management.

## Conclusion

When developing and implementing waitlist prioritization strategies, it is important to consider the resource constraints of the system and the patient profile, as the benefits of providing faster access to prioritized patients can lead to unreasonable wait-times for non-prioritized ones. In settings with long wait-times, prioritization initiatives must be followed by expansion of supply to achieve optimal improvements in health outcomes.

## Supplementary Material

qcae059_Supplemental_File

## Data availability

The data underlying this article cannot be shared publicly due to privacy reasons.
