# Supplementary material for: Wait-times benchmarks for risk-based prioritization in transcatheter aortic valve implantation: a simulation study
Source: Eur Heart J Qual Care Clin Outcomes. 2024 Jul 19;11(1):10–8. doi: 10.1093/ehjqcco/qcae059 (PMC11736150; doi:10.1093/ehjqcco/qcae059)
Supplement: qcae059_Supplemental_File [file qcae059_supplemental_file.docx]

# wait-times Benchmarks for risk-based prioritization in Transcatheter Aortic Valve Implantation: a simulation study

# Supplemental material

# Results, sensitivity analysis

When we divided the cohort into 3 equally sized risk groups, wait-times in the low-risk group were longer but health outcomes were better, compared to the combinations of the main analysis (figure S2). Waitlist mortality in the low-risk group varied between 2.4% and 2.81% (RR 0.67 and 0.78), waitlist hospitalization between 24.45% and 28.39% (RR 0.73 and 0.85), the proportion of urgent TAVI between 8.75% and 10.75% (RR 0.58 and 0.71), and wait-times in the low-risk group varied between 21.5 and 29.5 weeks. There were no combinations with wait-times in the low-risk group ≤16 weeks.

In the scenario with 2 risk groups instead of 3 (figure S3), the wait-times in the low-risk group were the lowest among all the simulations, varying between 12.9 and 16.6 weeks. On the other hand, we observed the lowest improvement in health outcomes when compared to the combinations of the main analysis: waitlist mortality between 2.87% and 3.38% (RR 0.8 and 0.94), waitlist hospitalization between 28.69% and 32.36% (RR 0.86 and 0.97), and proportion of urgent TAVI between 10.99% and 13.64% (RR 0.72 and 0.9). In most of the combinations in this scenario the wait-times wait-times in the low-risk group were ≤16 weeks (fig S4E-H).

### **Figure S1.** Model schematic


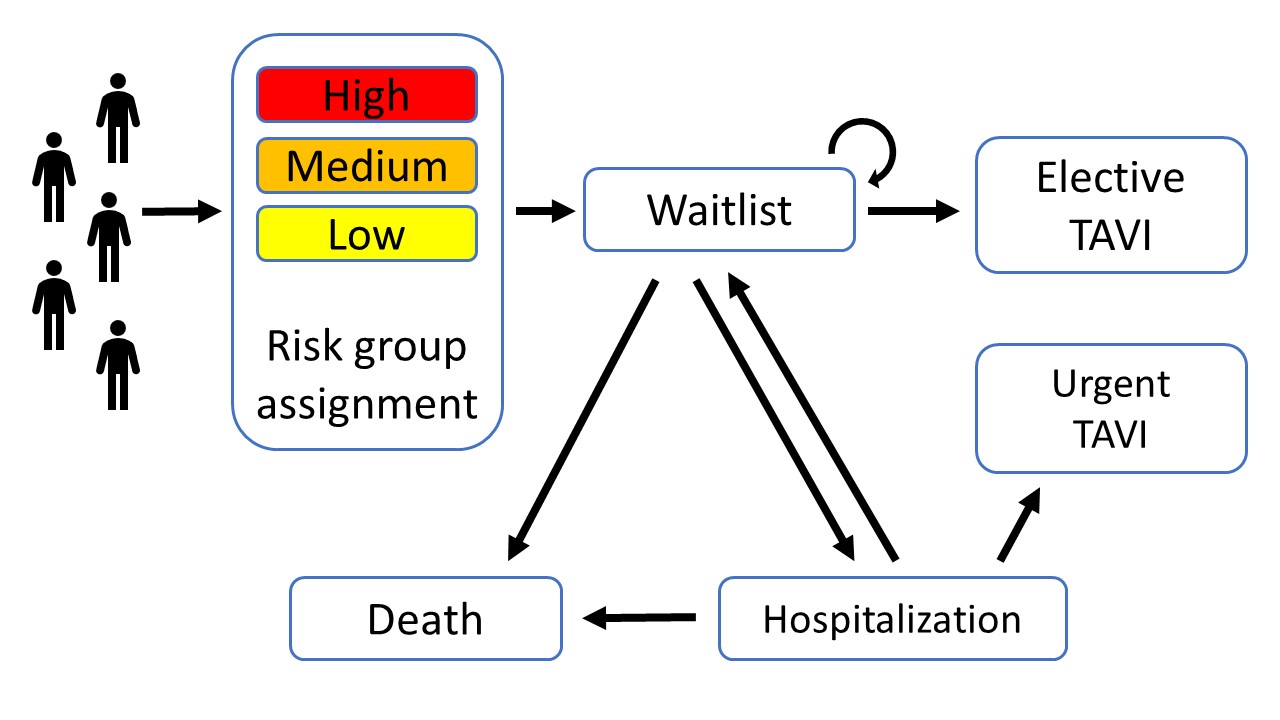


### **Figure S2.** Impact of size and wait-times of the high- and medium-risk groups. Analysis with 3 groups of equal sizes

1. Impact on mortality (%)


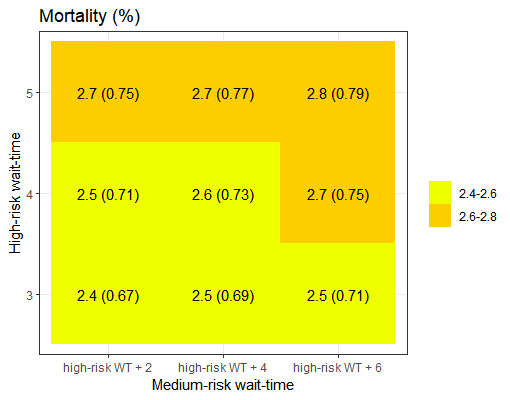


1. Impact on hospitalizations (%)


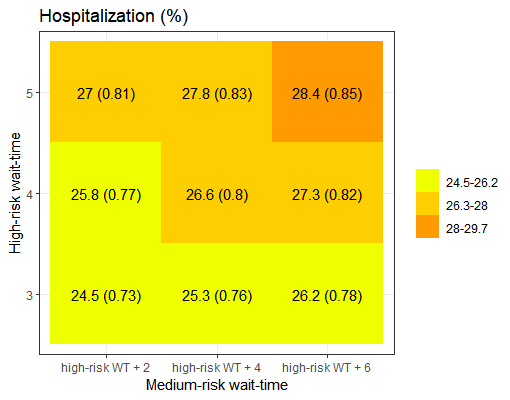


1. Impact on proportion of urgent TAVIs (%)


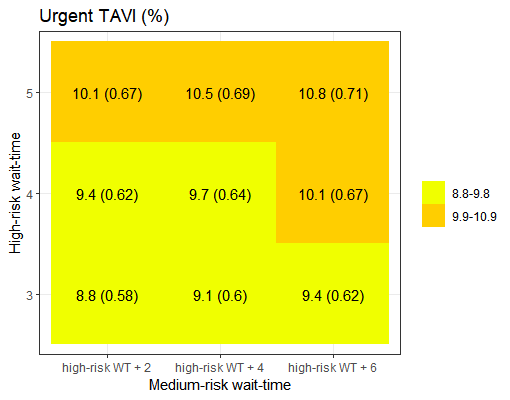


1. Impact on wait-times in the low-risk group (weeks)


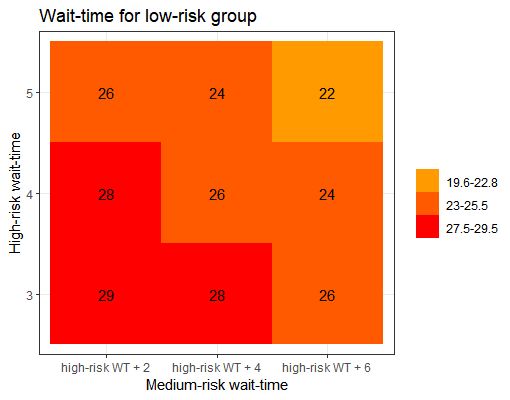


WT: wait-time

### **Figure S3.** Impact of size and wait-times of the high-risk groups. Analysis with 2 risk groups

1. Impact on mortality (%)


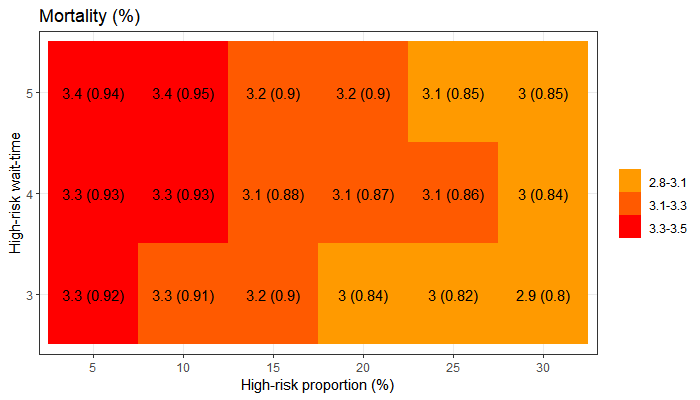


1. Impact on hospitalizations (%)


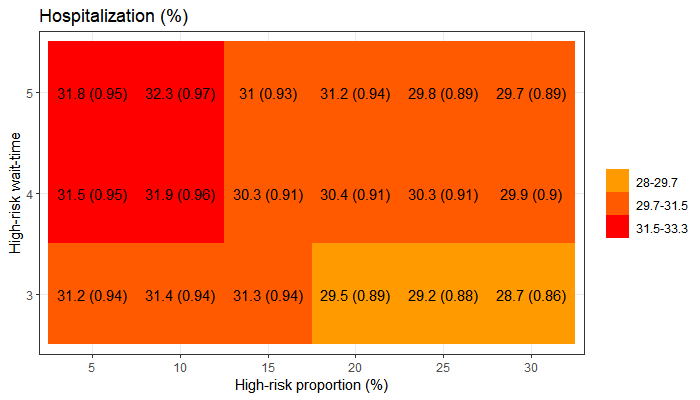


1. Impact on proportion of urgent TAVIs (%)


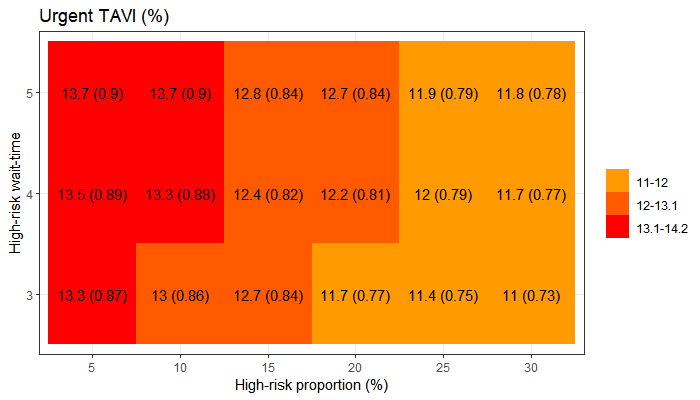


1. Impact on wait-times in the low-risk group (weeks)


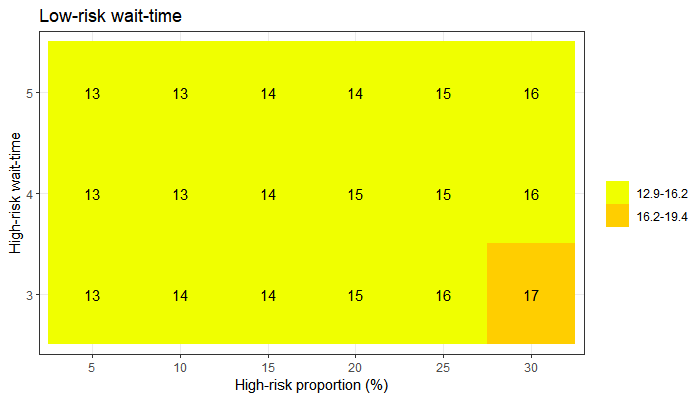


### **Figure S4.** Figures 1-4, S3 after removal of combinations in which wait-times in the low-risk group were greater then 16 weeks

1. Impact of size and wait-times of the high- and medium-risk groups on mortality (%) (figure 1)


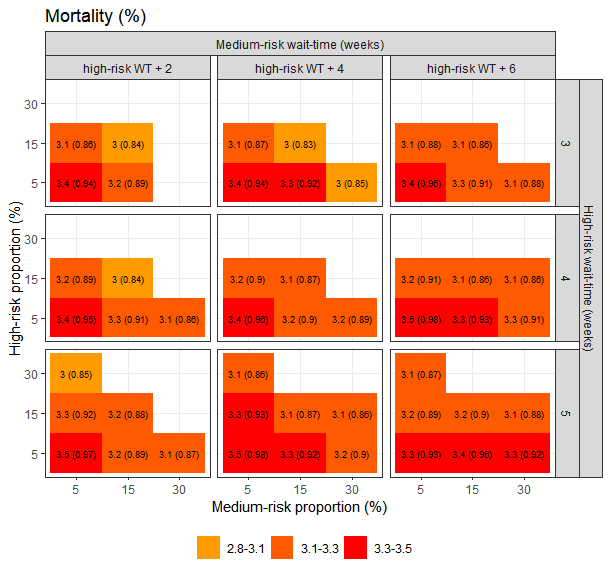


1. Impact of size and wait-times of the high- and medium-risk groups on hospitalizations (%) (figure 2)


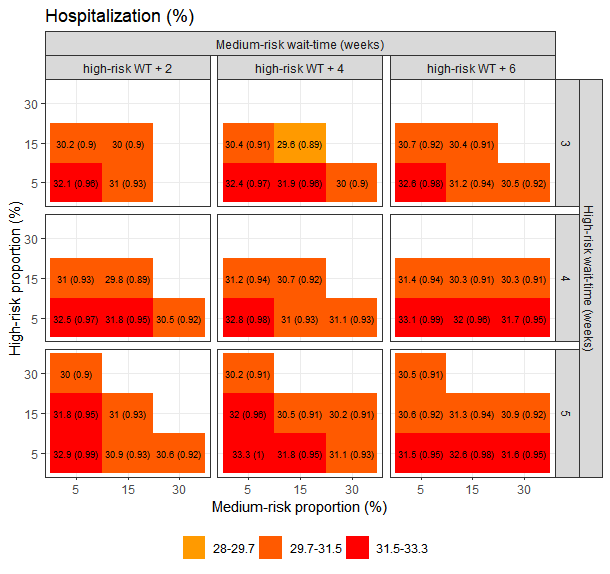


1. Impact of size and wait-times of the high- and medium-risk groups on proportion of urgent TAVIs (%) (figure 3)


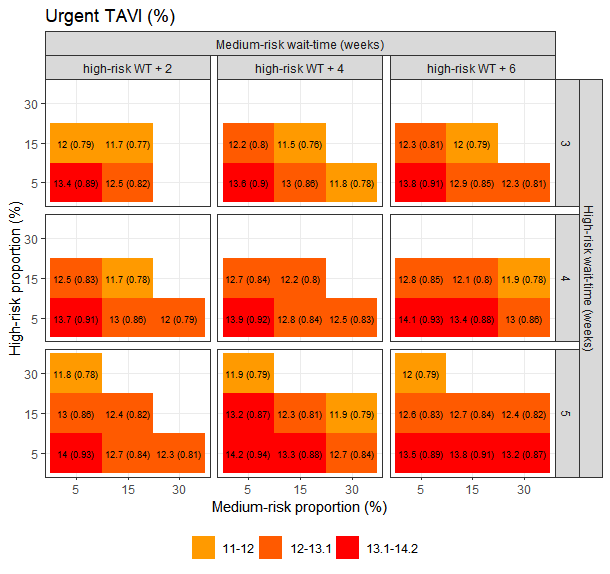


1. Impact of size and wait-times of the high- and medium-risk groups on wait-times in the low-risk group (weeks) (figure 4)


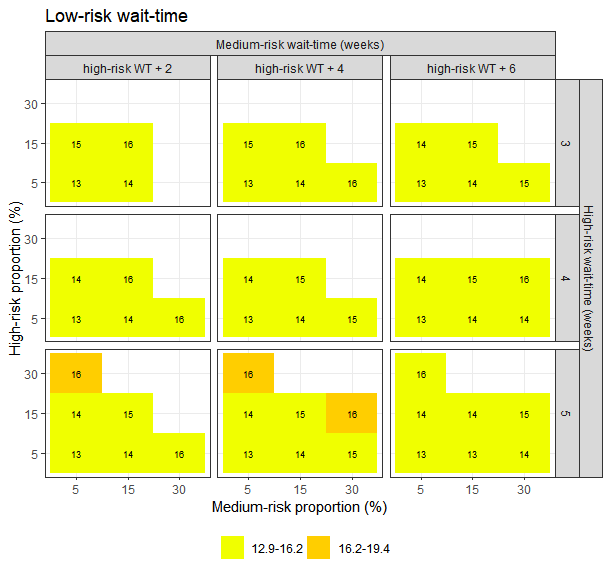


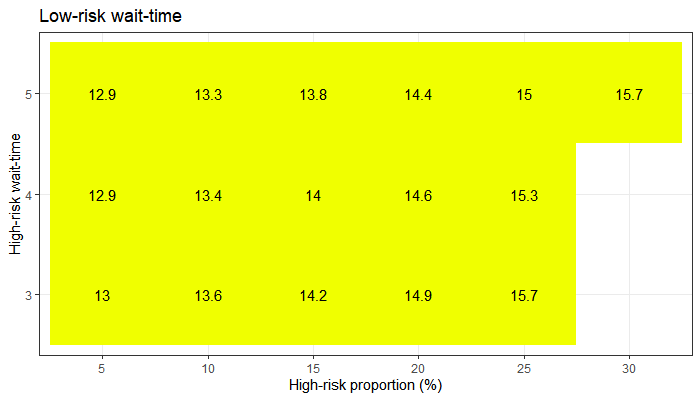


1. Impact on mortality (%) (figure S3A)


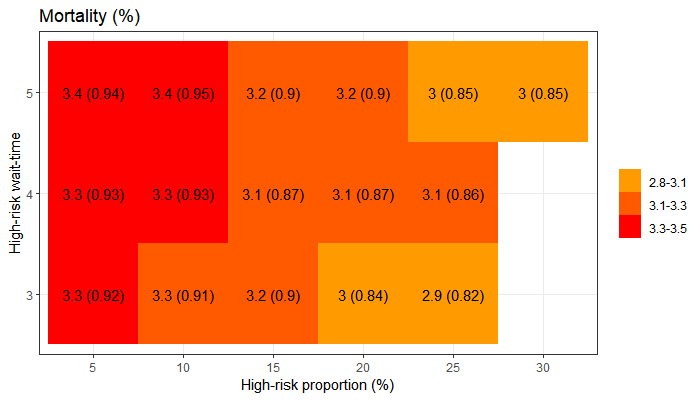


1. Impact Impact on hospitalizations (%) (figure S3B)


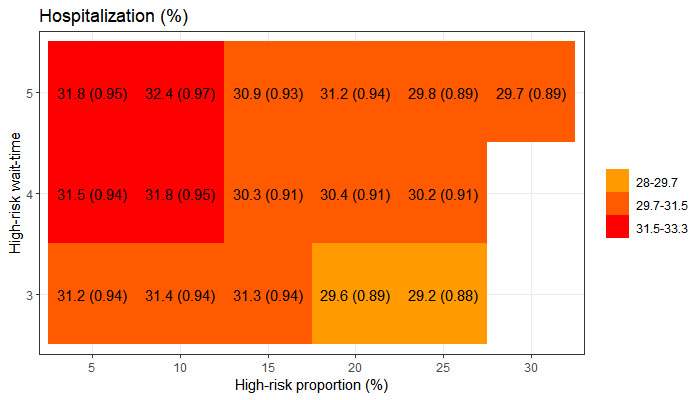


1. Impact on proportion of urgent TAVIs (%) (figure S3C)


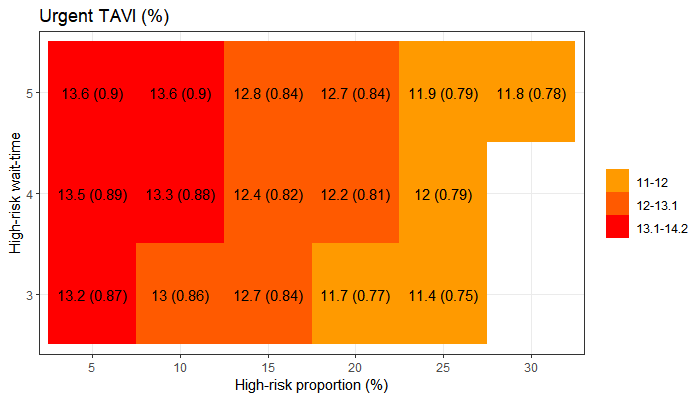


1. Impact on wait-times in the low-risk group (weeks) (figure S3D)


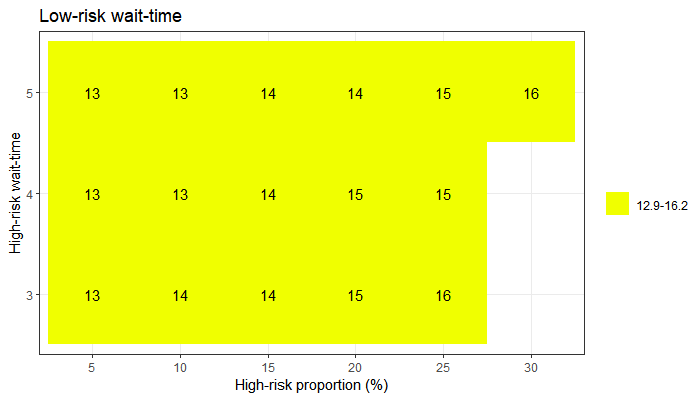


# Tables

**Table S1:** Model input parameters

| **Parameter (distribution)** | **Value** |
| --- | --- |
| Distribution of predicted risk of death (lognormal) | Mean (log) = -3.68  SD (log) = 0.5 |
| Distribution of predicted risk of hospitalization (lognormal) | Mean (log) = -1.22  SD (log) = 0.33 |
| Distribution of predicted risk of urgent TAVI (lognormal) | Mean (log) = -2.5  SD (log) = 0.65 |
| Cycle-specific probability of death (Weibull) | Shape = 1.0533  Scale = variable |
| Cycle-specific probability of hospitalization (Weibull) | Shape = 1.1267  Scale = variable |
| Overall mortality | 3.64%* |
| Overall hospitalization | 33.95%* |
| Overall proportion of urgent TAVI | 15.19%* |
| Overall wait-time for TAVI | 12.5 weeks |

*Targets used in calibration

Table S2. Distributions of predicted risks at 12 weeks

| **Percentile** | **All-cause mortality** | **All-cause hospitalization** | **Urgent TAVI** |
| --- | --- | --- | --- |
| Minimum | 0.0063 | 0.096 | 0.0182 |
| 5 | 0.0117 | 0.1617 | 0.0245 |
| 10 | 0.0125 | 0.1848 | 0.0365 |
| 15 | 0.0137 | 0.1989 | 0.0365 |
| 20 | 0.0152 | 0.2127 | 0.0418 |
| 25 | 0.0159 | 0.228 | 0.0523 |
| 30 | 0.0167 | 0.2429 | 0.058 |
| 35 | 0.0177 | 0.2542 | 0.0664 |
| 40 | 0.0203 | 0.2655 | 0.0725 |
| 45 | 0.0227 | 0.2756 | 0.0725 |
| 50 | 0.0247 | 0.2876 | 0.078 |
| 55 | 0.0268 | 0.303 | 0.0828 |
| 60 | 0.0295 | 0.3217 | 0.102 |
| 65 | 0.0314 | 0.3392 | 0.114 |
| 70 | 0.0332 | 0.3553 | 0.114 |
| 75 | 0.0349 | 0.3694 | 0.1298 |
| 80 | 0.0374 | 0.3842 | 0.1298 |
| 85 | 0.0409 | 0.4028 | 0.151 |
| 90 | 0.045 | 0.4277 | 0.1886 |
| 95 | 0.0537 | 0.4728 | 0.2333 |
| Maximum | 0.2387 | 0.6933 | 0.4693 |
